# Apparent diffusion coefficient measurement by diffusion weighted magnetic resonance imaging is a useful tool in differentiating renal tumors

**DOI:** 10.1186/s12885-015-1221-1

**Published:** 2015-04-16

**Authors:** Jing-Hong Liu, Shi-Feng Tian, Ye Ju, Ye Li, An-Liang Chen, Li-Hua Chen, Ai-Lian Liu

**Affiliations:** 1 Department of Radiology, The First Affiliated Hospital of Dalian Medical University, Zhongshan Road No. 222, Xigang District, Dalian, 116011 P.R. China; 2 Department of Radiology, Dalian Medical University, Dalian, 116044 P.R. China

**Keywords:** Diffusion weighted magnetic resonance imaging, Apparent diffusion coefficient, Renal tumors, Differentiation, B value, Renal malignant tissues, Benign renal tumor tissues, Meta-analysis

## Abstract

**Background:**

To determine the clinical value of apparent diffusion coefficient (ADC) measurement by diffusion weighted magnetic resonance imaging (DW-MRI) in differentiating renal tumors.

**Methods:**

Electronic databases were searched using combinations of keywords and free words relating to renal tumor, ADC and DW-MRI. Based on carefully selected inclusion and exclusion criteria, relevant case–control studies were identified and the related clinical data was acquired. Statistical analyses were performed using STATA 12.0 (Stata Corporation, College station, TX).

**Results:**

Sixteen case–control studies were ultimately included in the present meta-analysis. These 16 high quality studies contained a combined total of 438 normal renal tissues and 832 renal tumor lesions (597 malignant and 235 benign). The results revealed that ADC values of malignant renal tumor tissues were markedly lower than normal renal tissues and benign renal tumor tissues. **A**DC values of benign renal tumor tissues were also significantly lower than normal renal tissue.

**Conclusions:**

ADC measurement by DW-MRI provided clinically useful information on the internal structure of renal tumors and could be an important radiographic index for differentiation of malignant renal tumors from benign renal tumors.

**Electronic supplementary material:**

The online version of this article (doi:10.1186/s12885-015-1221-1) contains supplementary material, which is available to authorized users.

## Background

Kidney cancers in adults involve malignant tumors originating from renal pelvis and renal parenchyma [1]. It is the deadliest of urological malignancies, with an estimated 58,000 Americans diagnosed in 2010 alone, and is associated with a relatively poor five-year survival rate of 65% [2]. Clinically, nearly 80-90% of kidney cancers are classified as renal cell carcinoma (RCC), which arises in the renal parenchyma [3,4]. RCC accounts for 2-3% of all malignancies in adults, and is the seventh most frequent cancer in men and the ninth most frequent cancer in women [5]. Although the overall survival rate is more than 60% over 5 years, approximately 30% of RCC patients diagnosed with a localized disease at presentation will progress to develop metastatic disease [6]. Etiologically, the established risk factors for RCC include genetic component (such as the von-Hippel Lindau gene mutations), race (African Americans have higher incidence), gender (higher risk in males), obesity, smoking and hypertension [1,7]. Recently, owing to the rampant use of abdominal imaging techniques in clinical diagnostics, such as ultrasonography, computed tomography and magnetic resonance imaging, the proportion of small and incidental renal tumors have increased sharply [5]. The best chance to cure RCC is through nephrectomy, and given that RCC is refractive to chemotherapy and radiation therapy, early diagnosis is currently the best approach to increasing patient survival [8]. Certain lesions may imitate tumors on diagnostic imaging, even though lesions are histologically composed of normal or benign renal tissues [9]. In clinical practice, a clear differentiation between benign and malignant renal tumors is critical for therapy planning and to distinguish surgical from non-surgical tumors [10]. Despite the technological advances in diagnostic imaging, the possibility of surgical discovery of benign pathology remains high in suspected cases of renal malignancy [9].

Diffusion-weighted magnetic resonance imaging (DW-MRI) measures the Brownian motion of water molecules in biological tissues [11]. It is sensitive to random motion of endogenous water molecules within the tissue environment, and the technique is particularly suited to both clinical and basic science applications [12]. DW-MRI provides critical information on biophysical properties of tissues, such as cell organization, cell density, microstructure and microcirculation, via detecting the motion of water molecules within a voxel without the need for administration of contrast agents [13]. The apparent diffusion coefficient (ADC) is a quantitative parameter computed from DW-MRI data to assess the extent of diffusion of water molecules [14]. Increased cellular density limits water diffusion into the interstitial space and ADC values are inversely proportional to cell density, thus ADC values are useful to obtain clinically useful correlations in a disease setting [10]. For instance, ADC values provide a non-invasive method to predict the histological subtype and nuclear grade of RCC [15]. Recently, application of DW-MRI in oncology imaging has improved the differential diagnosis of benign and malignant tumors in brain, liver, breast, prostate and several female pelvic organs [16-19]. However, few reports discuss the clinical value of DW-MRI in differentiating between renal tumors [20,21]. To address this issue, we performed a comprehensive meta-analysis to examine published data for assessment of the clinical value of ADC measurement by DW-MRI in differentiation of renal tumors.

## Methods

### Search strategy

A systematic search of electronic databases, including PubMed, Wiley, EBSCO, SpringerLink, Web of Science, Ovid, China National Knowledge Infrastructure (CNKI), Wanfang database and VIP Information databases, was performed (last search in October 2014), according to the PRISMA guidelines (http://prisma-statement.org/, as shown in Additional file 1). Random combinations of following keywords was utilized for the search: (“diffusion magnetic resonance imaging” or “diffusion MRI” or “diffusion weighted MRI” or “diffusion weighted imaging” or “DWI” or “WB-DWI” or “DMRI” or “diffusion ”), (“kidney neoplasms” or “renal neoplasms” or “cancer of kidney” or “kidney cancers” or “renal cancer” or “cancer of the kidney” or “renal adenocarcinoma” or “renal tumor” or “renal carcinoma” or “malignant tumor of kidney”). The title and abstract of studies retrieved from the search were examined manually to exclude inappropriate publication, and cross-references of all remaining literature on the study topic were inspected for additional relevant studies.

### Eligibility criteria

The studies selected in this meta-analysis were clinical case–control studies reporting differentiation of renal tumors using ADC measurement by DW-MRI. The eligible studies met the following inclusion criteria: (1) included study subjects were patients with renal tumors and healthy controls; (2) included papers provided complete data on age, country, language, ethnicity, gender, number of lesions, pathological types, types of MRI machines, b-value, and ADC value; (3) if multiple studies included overlapping data, only the study with latest or largest data was included.

Studies were excluded if (1) the research topic was unrelated to the differential diagnosis of renal tumors using ADC measurement by DW-MRI; (2) no comparisons between healthy controls and malignant tumors, or between benign tumor and malignant tumor; (3) studies published in languages other than Chinese or English; (4) repeat publications; (5) incomplete data.

### Data extraction and quality evaluation

Data from qualified studies were collected by two independent investigators, using a predefined data collection table. The predefined tables were designed to extract all relevant data, figures and tables from the texts, including country, first author, year, language, ethnicity, study design, patient number, age, gender, and pathological types.

Quality evaluation of included studies was performed by more than 2 investigators by methodological index for non-Randomized studies (MINORS) criteria [22]. MINORS, a verified scoring tool for non-randomized studies, include a 12-item assessment. The score of each item ranges from 0 to 2 with an ideal total score of 24 for comparative studies and a score of 16 for non-comparative studies. The specific 12 criteria were as follows: whether the stated aim was clear (MINORS01), whether the inclusion of patients was consecutive (MINORS02), whether the prospective data was collected (MINORS03), whether the endpoints for aim were appropriate (MINORS04), whether assessment of endpoint was unbiased (MINORS05), whether the follow-up period was appropriate (MINORS06), whether the loss to follow-up was less than 5% (MINORS07), whether study size was prospectively calculated (MINORS08), whether the control group was adequate (MINORS09), whether the groups were contemporary (MINORS10), whether the baseline of groups was equivalent (MINORS11), whether the statistical analyses were adequate (MINORS12).

### Statistical analysis

STATA 12.0 software (Stata Corporation, College Station, TX, USA) was used for statistical analysis in the present meta-analysis. The correlation between ADC measurement by DW-MRI and the differentiation of renal tumors was calculated by standard mean difference (SMD) with 95% confidence intervals (CI), applying a random-effects model or a fixed-effects model. The Z test was performed to determine the significance of pooled SMDs. Heterogeneity across studies was evaluated by Cochran’s Q-statistic (a *P* value < 0.05 was considered significant) and *I*^*2*^ test (0%, no heterogeneity; 100%, maximal heterogeneity) [23]. A random or fixed-effects model was used on the basis of the heterogeneity analysis. When significant heterogeneity existed among studies (*P* < 0.05 or *I*^*2*^ > 50%), a random-effects model was used, otherwise, a fixed-effects model was used [24,25]. Sensitivity analysis was performed by deleting single study one by one, to evaluate the effects of single study on the overall result. The publication bias which assessed the reliability of result was evaluated by contour-enhanced funnel plot and Egger test [26,27]. Univariate and multivariate meta-regression analyses were applied to examine the source of heterogeneity, and Monte Carlo simulation (MCS) was applied to correct and verify the results [28].

## Results

### Study selection

**Figure 1:**
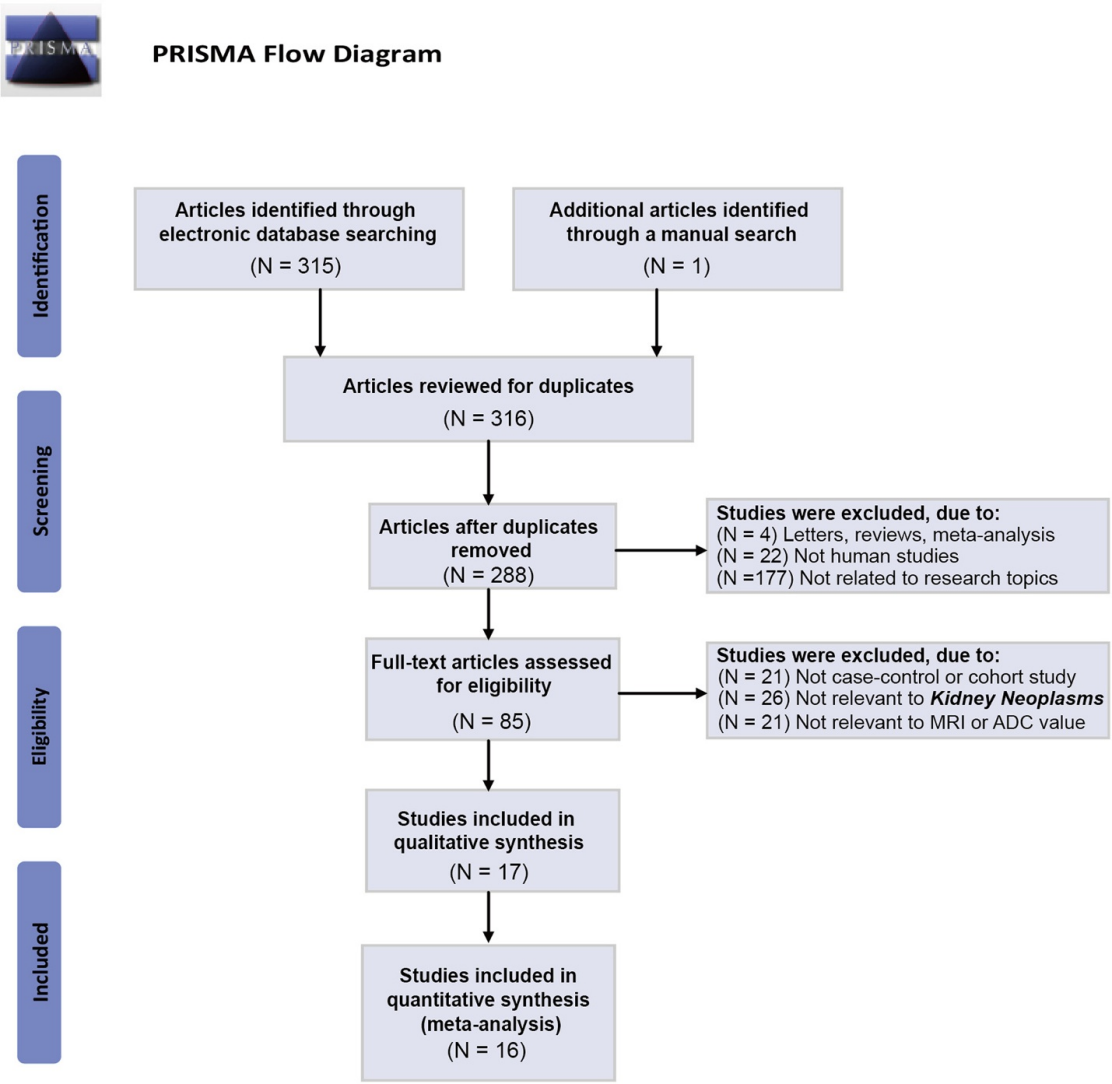
Flow chart of literature selection process.

**Figure 2:**
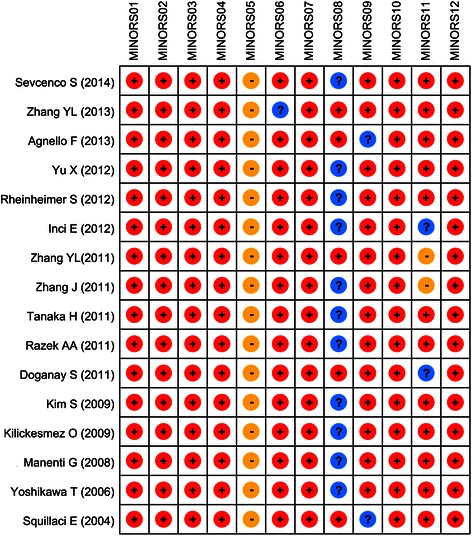
The quality scores of included studies by methodological index for non-randomized studies.

A total of 316 studies were retrieved after the search of electronic databases. Next, the articles were reviewed, resulting in 288 eligible articles after removing duplicates. After reading the full texts, we excluded 272 articles for the following reasons: the studies were not human studies (n = 22), were letters, reviews or meta-analyses (n = 4), were not related to research topics (n = 177), were not case–control studies (n = 21), were not relevant to kidney neoplasms (n = 26), were not relevant to MRI or ADC value (n = 21), and contained incomplete data in articles (n = 1). Sixteen articles [10,14,21,29-41] (14 in English and 2 in Chinese) satisfied the inclusion and exclusion criteria and were selected for data extraction and data analysis. Figure 1 shows the literature selection processes. All included studies were published between 2004 and 2014. Among the 16 studies, study subjects in 9 trials were Asians, 6 trials were performed in Caucasians and 1 trial was conducted in African population. Based on the country of publication, 4 studies were from China, 3 from Turkey, 1 from US, 2 from Japan, 2 from Italy, and 1 each from Austria, France, Germany, Egypt. This meta-analysis included 438 normal healthy renal tissues and 832 renal tumor lesions (597 malignant and 235 benign). The three types of MRI machines used were Siemens, GE and Philips, and the b-values were 500 s/mm^2^, 600 s/mm^2^, 800 s/mm^2^, 1000 s/mm^2^, 500/1000 s/mm^2^ and 400/800 s/mm^2^. The quality score and the baseline characteristics of included studies are shown in Figure 2 and Table 1, respectively.

**Table 1:** **Baseline characteristics of the sixteen included studies**

First author	Year	Country	Ethnicity	Age (years)	Gender (F/M)	MRI machine type	b value (s/mm^2^)	b value (s/mm^2^)
Sevcenco S [21]	2014	Austria	Caucasians	64(21–85)	-	Siemens	500/1000	71
Zhang YL [29]	2013	China	Asians	52	41/23	GE	500	121
Agnello F [30]	2013	France	Caucasians	61.6(27–88)	19/16	Philips	1000	47
Yu X [31]	2012	China	Asians	53 (30–81)	93/44	GE	800	274
Rheinheimer S [32]	2012	Germany	Caucasians	60.4 (36–83)	37/19	Siemens	800	54
Inci E [33]	2012	Turkey	Asians	53.5	74/61	Siemens	500/1000	88
Zhang YL-a [34]	2011	China	Asians	7-79	-	Philips	500	97
Zhang YL-c [34]	2011	China	Asians	7-79	-	Philips	800	97
Zhang YL-d [34]	2011	China	Asians	7-79	-	Philips	1000	97
Zhang J [35]	2011	China	Asians	30-76	-	GE	800	40
Tanaka H[14]	2011	Japan	Asians	57 (38 ~ 78)	21/14	Philips	800	82
Razek AA [10]	2011	Egypt	Africans	5-67	24/28	GE	800	54
Doganay S-b [36]	2011	Turkey	Asians	53(1–76)	25/33	GE	600	117
Doganay S-d [36]	2011	Turkey	Asians	53(1–76)	25/33	GE	1000	117
Kim S [37]	2009	USA	Caucasians	-	-	Siemens	400/800	64
Kilickesmez O [38]	2009	Turkey	Asians	45.6	46/56	Siemens	500/1000	66
Manenti G [39]	2008	Italy	Caucasians	58.8(30–85)	22/15	Philips	500	37
Yoshikawa T [40]	2006	Japan	Asians	61.9	122/78	Philips	600	20
Squillaci E [41]	2004	Italy	Caucasians	55.7(29–85)	20/18	Philips	500	38

F = female; M = male; MRI = magnetic resonance imaging; GE = general electric; a, b = 500; b, b = 600; c, b = 800; d, b =1000.

### Results of meta-analysis

**Figure 3:**
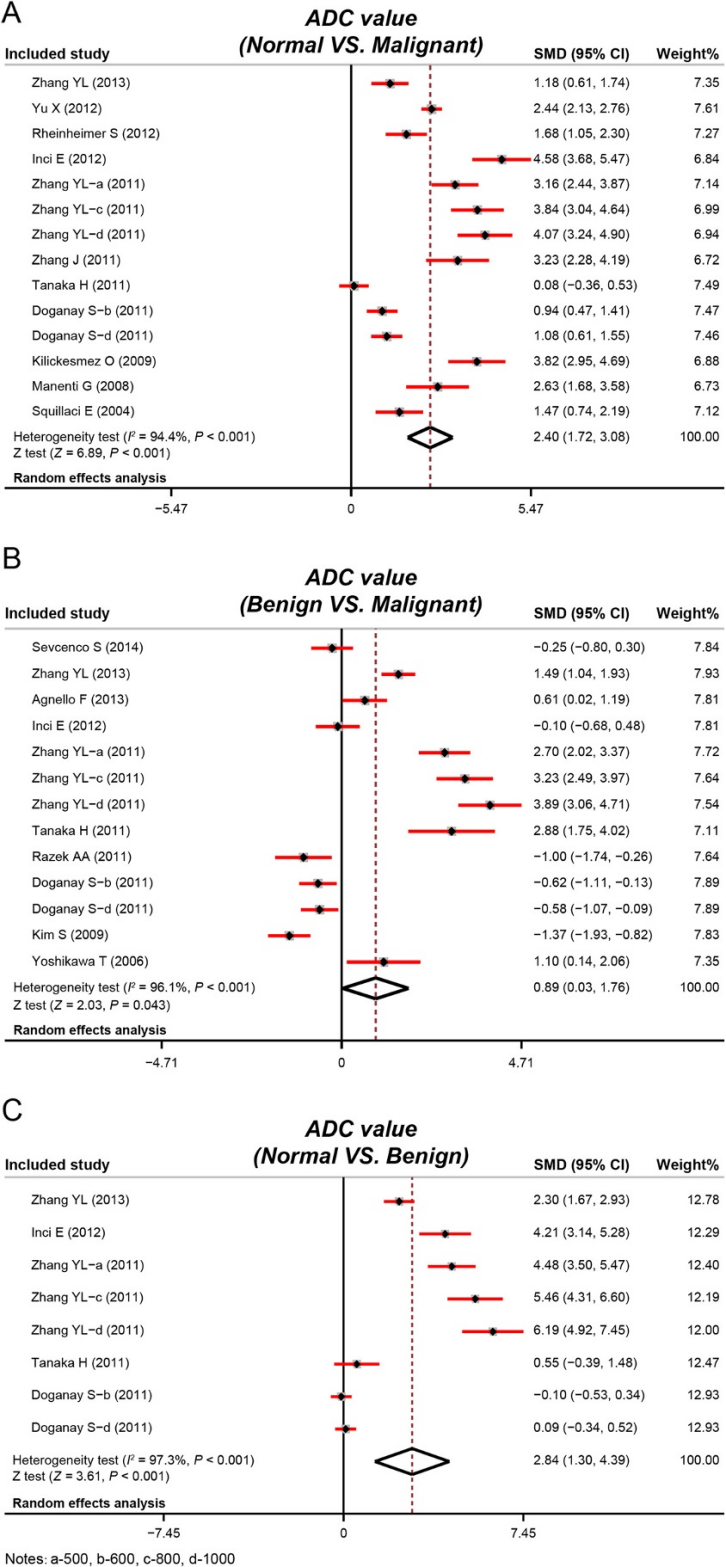
Forest plots of apparent diffusion coefficient values of diffusion-weighted magnetic resonance imaging in differentiation of renal tumors (**A**: comparison of ADC values between normal renal tissues and malignant renal tumor tissues; **B**: comparison of ADC values between benign and malignant renal tumor tissues; **C**: comparison of ADC values between normal renal tissues and benign renal tumor tissues).

The heterogeneity test revealed that there was heterogeneity across studies that compared ADC values between different tissues (normal renal tissues vs. malignant renal tumor tissues: *P* < 0.001, *I*^*2*^ = 94.4%; malignant renal tumor tissues vs. benign renal tumor tissues: *P* < 0.001, *I*^*2*^ = 96.1%; normal renal tissues vs. benign renal tumor tissues: *P* < 0.001, *I*^*2*^ = 97.3%), thus a random-effects model was applied in all cases. As shown in Figure 3, the present meta-analysis revealed that the ADC values of malignant renal tumor tissues were significantly lower than normal renal tissues (SMD = 2.40, 95% CI = 1.72 ~ 3.08, *P* < 0.001) and benign renal tumor tissues (SMD = 0.89, 95% CI = 0.03 ~ 1.76, *P* = 0.043). ADC values of benign renal tumor tissues were also significantly lower than normal renal tissues (SMD = 2.84, 95% CI = 1.30 ~ 4.39, *P* < 0.001).

### Subgroup analyses

Subgroup analysis based on the types of MRI equipment indicated that there was significant difference between ADC values of normal renal tissue vs. malignant renal tumor tissue, normal renal tissue vs. benign renal tumor tissue, and benign renal tumor tissue and malignant renal tumor tissue when the equipment used was Siemens and Philips (*P* < 0.05). Significant differences also existed in ADC values of normal renal tissue vs. malignant renal tumor tissue, when the MRI equipment was from GE (*P* < 0.05).

Additionally, subgroup analysis based on b-value found that when b = 500 s/mm^2^, 600 s/mm^2^, 800 s/mm^2^, 500/1000 s/mm^2^, there was significant difference between ADC values of normal renal tissue and malignant renal tumor tissue (*P* < 0.01), while at b = 1000 s/mm^2^, no significant difference was observed (*P* > 0.05). When b = 500 s/mm^2^, 500/1000 s/mm^2^, there was significant difference between ADC values of normal renal tissue and benign renal tumor tissue (*P* < 0.05), while at b = 600 s/mm^2^, 800 s/mm^2^, 1000 s/mm^2^, no marked difference was detected (*P* > 0.05). Specific results of subgroup analyses on ADC values of DW-MRI in differentiation of renal tumors are present in Table 2.

**Table 2:** **Standard mean difference of subgroup analyses on apparent diffusion coefficient values of diffusion-weighted magnetic resonance imaging in differential diagnosis of renal tumors**

	Normal VS. Malignant			Benign VS. Malignant			Normal VS. Benign		
	SMD	95% CI	*P*	SMD	95% CI	*P*	SMD	95% CI	*P*
Ethnicity:									
Asians	2.54	1.71-3.37	<0.001	0.22	−1.45-0.77	0.016	2.84	1.30-4.39	< 0.001
Caucasians	1.85	1.24-2.45	< 0.001	−1.07	0.43-2.62	0.013	-	-	-
Africans	-	-	-	−1.00	−1.74-(−0.26)	0.008	-	-	-
Machine type:									
GE	1.73	0.94-2.52	< 0.001	−0.05	−0.21-0.30	0.713	0.74	−0.55-2.04	0.259
SIEMENS	3.34	1.50-5.17	< 0.001	−0.58	−0.91-(−0.26)	< 0.001	4.21	3.14-5.28	< 0.001
PHILIPS	2.52	1.07-3.98	0.001	2.21	1.90-2.53	< 0.001	4.15	1.56-6.73	0.002
b value:									
500	2.08	1.11-3.05	< 0.001	1.85	1.48-2.22	< 0.001	3.36	1.22-5.49	0.002
600	0.94	0.47-1.41	< 0.001	−0.27	−0.70-0.17	0.233	−0.10	−0.53-0.34	0.664
800	2.22	0.97-3.48	0.001	1.43	0.95-1.90	< 0.001	2.99	−1.82-7.80	0.223
1000	2.55	−0.37-5.48	0.087	0.59	0.25-0.93	0.001	3.11	−2.87-9.08	0.308
400/800	-	-	-	−1.37	−1.93-(−0.82)	< 0.001	-	-	-
500/1000	4.19	3.45-4.93	< 0.001	−0.18	−0.58-0.22	0.375	4.21	3.14-5.28	< 0.001

SMD = standard mean difference; 95% CI = 95% confidence intervals; GE = general electric.

### Sensitivity analysis and publication bias

**Figure 4:**
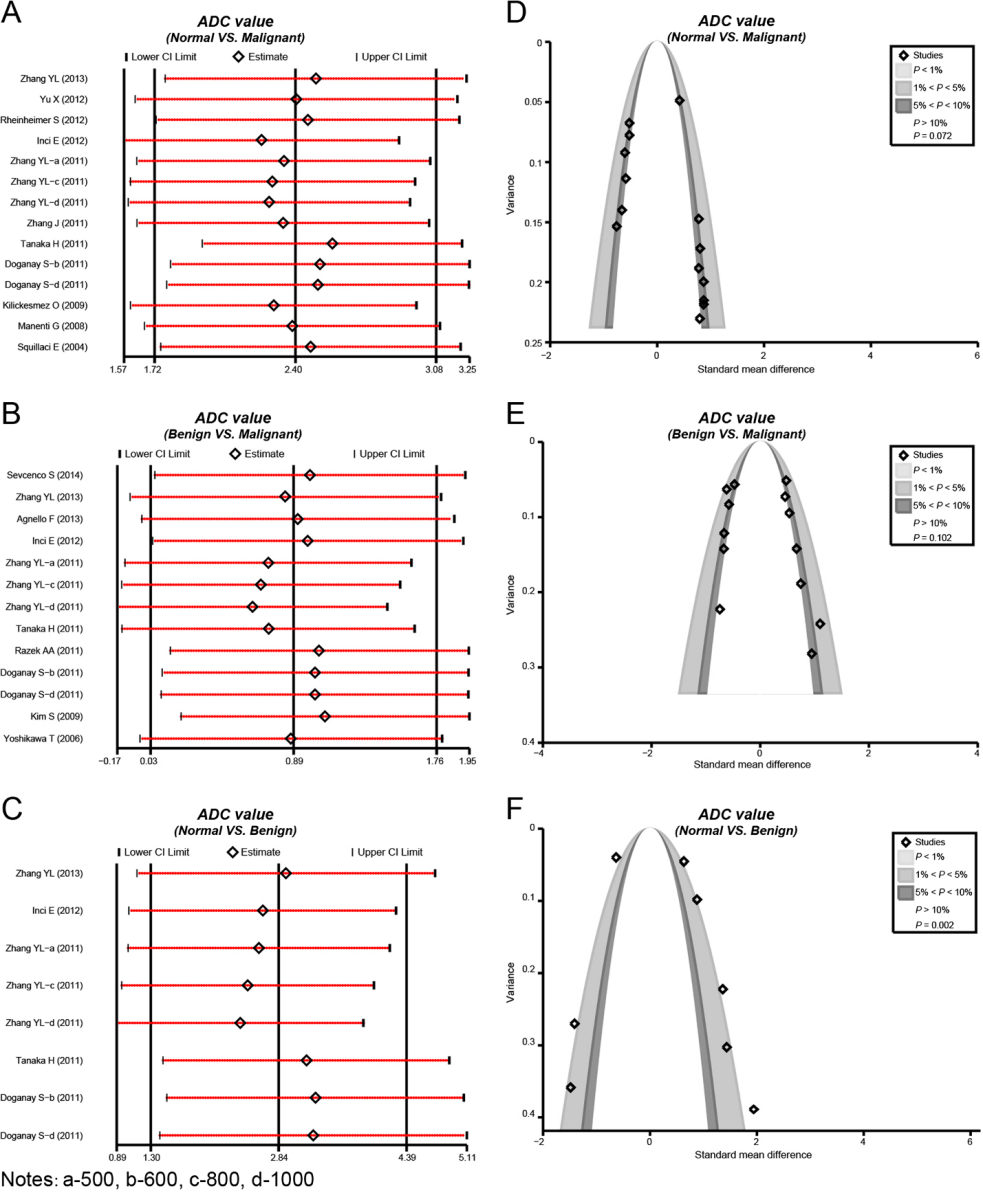
Sensitivity analyses and funnel plots of apparent diffusion coefficient values of diffusion-weighted magnetic resonance imaging in differentiation of renal tumors (**A**: Sensitivity analysis comparing normal renal tissues and malignant renal tumor tissues; **B**: Sensitivity analysis comparing benign and malignant renal tumor tissues; **C**: Sensitivity analysis comparing normal renal tissues and benign renal tumor tissues; **D**: Publication bias comparing normal renal tissues and malignant renal tumor tissues; **E**: Publication bias comparing benign and malignant renal tumor tissues; **F**: Publication bias comparing normal renal tissues and benign renal tumor tissues).

All studies related to comparisons between normal renal tissue and malignant renal tumor tissue, and the comparisons between malignant renal tumor tissue and benign renal tumor tissue showed no evident effect on the pooled SMD. The Contour-enhanced funnel plots, of studies investigating the comparisons between normal renal tissue and malignant renal tumor tissue as well as the comparisons between benign renal tumor tissue and malignant renal tumor tissue, indicated there was publication bias (*P* > 0.05) which was further confirmed by the Egger test (*P* > 0.05). There was no publication bias across studies that explored comparisons between normal tumor tissue and benign renal tumor tissue (*P* < 0.01), which was also confirmed by the Egger test (*P* = 0.002) (as shown in Figure 4).

### Regression analysis

Univariate meta-regression analysis and multivariate meta-regression analysis were conducted. Univariate meta-regression analysis showed that, in studies that carried out the comparisons between normal renal tissue and malignant renal tumor tissue, publication year, sample size, country, ethnicity, types of MRI mechanisms and b-value had no correlation with heterogeneity (*P* > 0.05), while language might be related to heterogeneity (*P* = 0.044). In studies that investigated comparisons between benign renal tumor tissue and malignant renal tumor tissue, publication year, sample size, ethnicity, b-value (*P* > 0.05) were not related to heterogeneity, and country, language and types of MRI machines correlate with heterogeneity (*P* < 0.05). In studies that carried out the comparisons between normal renal tissue and benign renal tumor tissue, country might be associated with heterogeneity (*P* < 0.05), but not publication year, sample size, publication year, and sample sizes (*P* > 0.05).

The multivariate meta-regression analysis revealed that, as shown in Table 3 and Table 4, in studies that compared normal renal tissue and malignant renal tumor tissue or between benign renal tumor tissue and malignant renal tumor tissue, publication year, sample size, country, language, ethnicity, types of MRI equipment and b value of MRI were not the main sources of heterogeneity. In studies that carried out the comparisons of normal renal tissue and benign renal tumor tissue, country was the main source of heterogeneity, but not the publication year, sample size, types of MRI machines or b-value (Table 5).

**Table 3:** **Multivariate regression analyses of apparent diffusion coefficient values in normal and malignant renal tumor tissues**

Heterogeneity factors	Coefficient	SE	t	*P* (Adjusted)	95% CI	
					LL	UL
Year	0.107	0.422	0.25	1	−0.925	1.138
Sample Size	0.005	0.112	0.45	0.994	−0.223	0.032
Country	0.18	0.54	0.33	0.998	−1.14	1.5
Language	2.372	2.667	0.89	0.878	−4.154	8.898
Ethnicity	−0.353	1.501	−0.24	1	−4.025	3.32
Machine type	−0.056	0.792	−0.07	1	−1.993	1.88
b value	−0.338	0.454	−0.74	0.941	−1.449	0.774

SE = standard error; 95% CI = 95% confidence intervals; LL = lower limit; UL = upper limit.

**Table 4:** **Meta-regression analyses of apparent diffusion coefficient values in malignant and benign renal tumor tissues**

Heterogeneity factors	Coefficient	SE	t	*P* (Adjusted)	95% CI	
					LL	UL
Year	−0.424	0.358	−1.18	0.771	−1.204	0.356
Sample Size	−0.003	0.025	−0.12	1	−0.058	0.052
Country	−0.461	0.315	−1.47	0.598	−1.147	0.224
Language	1.857	1.721	1.08	0.827	−1.895	5.608
Ethnicity	0.159	0.752	0.21	1	−1.48	1.8
Machine type	0.433	0.74	0.59	0.988	−1.18	2.047
b value	0.038	0.332	0.11	1	−0.686	0.761

SE = standard error; 95% CI = 95% confidence intervals; LL = lower limit; UL = upper limit.

**Table 5:** **Meta-regression analyses of apparent diffusion coefficient values in normal and benign renal tumor tissues**

Heterogeneity factors	Coefficient	SE	t	*P* (Adjusted)	95% CI	
					LL	UL
Year	−3.385	0.97	−3.49	0.114	−7.558	0.789
Sample Size	−0.364	0.07	−5.18	0.149	−0.667	−0.061
Country	−1.785	0.259	−6.89	0.026	−2.901	−0.67
Machine type	−5.645	1.444	−3.91	0.092	−11.856	0.567
b value	−0.383	0.263	1.46	0.492	−0.746	1.513

SE = standard error; 95% CI = 95% confidence intervals; LL = lower limit; UL = upper limit.

## Discussion

The diagnostic value of DW-MRI as a stand-alone approach in detailed characterization of renal tumors is controversial. Some clinicians consider DW-MRI as an effective diagnostic tool to differentiate benign from malignant tumors in multiple organs [16-19], but others are skeptical about the physics and the dynamics of DW-MRI in a tumor setting [20,21]. We investigated the clinical significance of DW-MRI using a meta-analysis based approach.

DW-MRI is a noninvasive imaging technique that is sensitive to thermally driven water molecule motion inside the body [42]. This random motion is frequently represented with a monoexponential model with ADC as its parameter [43]. ADC is a quantitative tool for multiple clinical applications and is important in differentiating benign from malignant lesions, evaluating tumor aggressiveness, performing early assessment of tumor response to therapy [44]. RCCs are classically classified into several representative subtypes including clear cell, chromophobe, and papillary RCCs on the basis of histological appearance and abnormal presence of genetic patterns, and clinical courses [7]. Fortunately, with the advantages of DW-MRI in differentiating RCCs from normal renal parenchyma, ADC value could be helpful in characterizing RCC subtypes [20,31,45]. Accurate estimation of ADC is pivotal in precise diagnosis, evaluation, and monitoring of human pathologies [44]. In this meta-analysis, we found that the ADC values of malignant renal tumor tissues were markedly lower than normal renal tissues and benign renal tumor tissues. Further, the ADC values of benign renal tumor tissues were also significantly lower than normal renal tissues. The images acquired by DW-MRI are constructed via quantifying the diffusion of water molecules in tissues and DWI uses differences in water motion to discriminate between tissues of varying cellularity [46,47]. In renal malignant lesions, diffusion is often restricted due to higher cellularity, tissue disorganization and decreased extracellular space, generating higher signal intensity on DW-MRI [48]. Histologically, RCC is composed of large tumor cells with abundant clear cytoplasm and very narrow intercellular space, which restrict water movement and resulted in low ADC values [49]. Therefore, ADC measurements using DW-MRI has been used as a surrogate marker for cellularity, to evaluate successful treatment and cell kill [50]. Additionally, RCC tumors are unique due to the presence of hemosiderin deposits, which help in distinguishing RCC tumors from other tumors [51]. A manuscript by Childs et al. revealed that in-phase signal loss, likely correlating with hemosiderin deposits, is observed in approximately 21% of renal masses and 42% of papillary RCC, suggesting that the paramagnetic effect of hemosiderin is responsible for the losses of in-phase signal intensity and intravoxel dephasing induced by T2 RCC, frequently observed in RCC tumors [52]. This susceptibility-induced intravoxel dephasing is predominant in DW-MRI of RCC tumors because larger intravoxel dephasing degree causes greater signal intensity loss [53]. Therefore, hemosiderin within renal tumors may lead to limited sensitivity of DW-MRI in diagnosis of malignant renal tumors, as observed in several studies. Moreover, when intravenous contrast cannot be administered, for instance in patients with end-stage renal disease, and heterogeneity between T1 and T2 is very important, and one MRI sequence alone cannot be relied upon to differentiate between benign and malignant tumors.

We also conducted subgroup analyses by types of MRI machines and b-value. Subgroup analysis by types of MRI suggested that Siemens and Philips MRI were more broadly applicable, owing to their clinical efficiency, compared to GE. Lastly, subgroup analysis based on b-value, showed that MRI machines at different b-values might differ in discriminating the renal tumors.

Certain limitations existed in the study design and should be considered. First, the number of patients in several included studies was relatively small, and the number of patients with renal lesions was also relatively small, which might reduce the reliability of the conclusions. Second, our meta-analysis was based on published studies, which tend to report positive or significant results, while studies with negative or insignificant results are not available for analysis. This might have led to a publication bias, which may have overestimated the results. In addition, this meta-analysis was restricted to studies published in English or Chinese, which might have introduced bias. Moreover, different RCC subtypes in the selected studies may have statistically significant differences in ADC values, and might influence the final results of this meta-analysis to some extent.

## Conclusion

In conclusion, in spite of the limitations of our meta-analysis, the evidence supports that ADC measurement by DW-MRI is a useful tool to measure the properties of the internal structure of tumors, and could be an important radiographic index for the differentiation of renal tumors.

## Additional file

Additional file 1:
**PRISMA Checklist_1.**

## Abbreviations

ADC: Apparent diffusion coefficient
DW-MRI: Diffusion-weighted magnetic resonance imaging
RCC: Renal cell carcinoma
CNKI: China national knowledge infrastructure
MINORS: Methodological index for non-Randomized studies
SMD: Standard mean difference
CI: Confidence intervals
